# Target Recognition of SAR Images via Matching Attributed Scattering Centers with Binary Target Region

**DOI:** 10.3390/s18093019

**Published:** 2018-09-10

**Authors:** Jian Tan, Xiangtao Fan, Shenghua Wang, Yingchao Ren

**Affiliations:** 1 Hainan Key Laboratory of Earth Observation, Sanya 572029, China; tanjian@radi.ac.cn (J.T.); fanxiangtao_radi@163.com (X.F.); 2 Key Laboratory of Digital Earth Science, Institute of Remote Sensing and Digital Earth, Chinese Academy of Sciences, Beijing 100094, China; ryc_radi@163.com; 3 School of Public Administration and Mass Media, Beijing Information Science and Technology University, Beijing 100093, China

**Keywords:** synthetic aperture radar (SAR), target recognition, attributed scattering center (ASC), region matching, score fusion

## Abstract

A target recognition method of synthetic aperture radar (SAR) images is proposed via matching attributed scattering centers (ASCs) to binary target regions. The ASCs extracted from the test image are predicted as binary regions. In detail, each ASC is first transformed to the image domain based on the ASC model. Afterwards, the resulting image is converted to a binary region segmented by a global threshold. All the predicted binary regions of individual ASCs from the test sample are mapped to the binary target regions of the corresponding templates. Then, the matched regions are evaluated by three scores which are combined as a similarity measure via the score-level fusion. In the classification stage, the target label of the test sample is determined according to the fused similarities. The proposed region matching method avoids the conventional ASC matching problem, which involves the assignment of ASC sets. In addition, the predicted regions are more robust than the point features. The Moving and Stationary Target Acquisition and Recognition (MSTAR) dataset is used for performance evaluation in the experiments. According to the experimental results, the method in this study outperforms some traditional methods reported in the literature under several different operating conditions. Under the standard operating condition (SOC), the proposed method achieves very good performance, with an average recognition rate of 98.34%, which is higher than the traditional methods. Moreover, the robustness of the proposed method is also superior to the traditional methods under different extended operating conditions (EOCs), including configuration variants, large depression angle variation, noise contamination, and partial occlusion.

## 1. Introduction

Owing to the merits of synthetic aperture radar (SAR), interpreting high-resolution SAR images is becoming an important task for both military and civilian applications. As a key step of SAR interpretation, automatic target recognition (ATR) techniques are employed to decide the target label in an unknown image [1]. Typically, a general SAR ATR method is comprised of two parts: feature extraction and a decision engine. The former tries to obtain low-dimensional representations from the original images while conveying the original discrimination capability. In addition, the high dimensionality of the original image is reduced significantly, which helps improve the efficiency of the following classification. Different kinds of features are adopted or designed for SAR target recognition in the previous literature. The features describing the physical structures or shape of the target are extracted for SAR ATR, e.g., binary target region [2,3,4], target outline [5,6], target’s radar shadow [7], local texture [8], etc. Park et al. [2] design several descriptors from the binary target region for SAR target recognition. In [3], a SAR ATR method is designed through the matching of binary target regions, where the region residuals are processed by the binary morphological operations to enhance divergences between different classes. In [4], the binary target region is first described by the Zernike moments, and a support vector machine (SVM) is employed for classification afterwards. The target outline is taken as the discriminative feature in [5], which is approached by the Elliptical Fourier Series (EFS). Then, SVM is used to classify the outline descriptors. Yuan et al. use the local gradient ratio pattern to describe SAR images with application to target recognition [8]. The projection features are also prevalent in SAR ATR. Principal component analysis (PCA) [9], linear discriminant analysis (LDA) [9], and non-negative matrix factorization (NMF) [10] are often used to extract the projection features. Based on the idea of manifold learning, other projection features are designed to exploit the properties of the training samples [11,12,13]. In the high frequency area, the total backscattering of a whole target can be regarded as the summation of several individual scattering centers [14]. In this way, the scattering center features are discriminative for SAR target recognition. Several SAR ATR methods have been proposed using the attributed scattering centers (ASCs) which achieve good effectiveness and robustness [15,16,17,18,19]. In the classification stage, the classifiers (decision engines) are adopted or designed according to the properties of the extracted features. For features with unified forms, e.g., feature vectors extracted by PCA, classifiers like SVM [4,5,20,21], adaptive boosting (AdaBoost) [22], sparse representation-based classification (SRC) [21,23,24], etc., can be directly used for classification tasks. The deep learning method, i.e., convolution neural network (CNN), is also demonstrated to be notably effective for image interpretation [25,26,27,28,29]. In CNN, the hierarchical deep features are learned by the convolution layers with a softmax classifier to perform the multi-class regression at the end. However, for features with no specific orders, e.g., ASCs, the former classifiers cannot be directly employed for classification. Usually, a similarity measure between these features is defined [16,17,18]. Afterwards, the target label is assigned as the template class achieving the maximum similarity.

This paper proposes an efficient and effective method for SAR ATR via matching ASCs with binary target regions. In previous works [16,17,18] using ASCs for SAR ATR, a complex one-to-one correspondence is often built for the following similarity evaluation. In [16], Chiang et al. solve the assignment problem between two ASC sets using the Hungarian algorithm and evaluate the similarity as the posterior probability. Ding et al. exploit the line and triangle structures in the ASC set during the similarity evaluation based on the one-to-one correspondences between two ASC sets [17,18]. However, it is still a difficult and complex task to precisely build the correspondence between the ASCs for the following reasons [30]. First, there are always missing or false alarms caused by the extended operating conditions (EOCs) such as occlusion, noises, etc. Second, the ASCs cannot be extracted with no errors. As a result, the extraction errors also cause problems. Lastly, as point features, the ASCs lack of high stability, especially because SAR images change greatly with variations in the target azimuth [31]. As a remedy, in this study, each of the extracted ASCs from the test image is represented by a binary region. In detail, the backscattering field of the ASC is first calculated based on the ASC method, and then transformed to the image domain. Afterwards, a global threshold is used to segment the reconstructed images of individual ASCs as binary regions. In the image domain, the spatial positions of the ASCs can be intuitively observed. For ASCs with higher amplitudes, they tend to produce regions with larger areas because their images contain more pixels with high intensities. In addition, the distributed ASCs with lengths could also maintain their attributes at proper thresholds. Hence, the predicted binary regions actually embody the attributes of the ASCs such as the spatial positions, relative amplitudes, and lengths. The binary regions of individual ASCs are matched to the extracted binary target region from the corresponding template samples. The overlap and differences during the region matching reflect the correlations between the test image and corresponding templates from various classes. Based on the region matching results, three matching scores are defined. To combine the strengths of different scores, a score-level fusion is performed to obtain a unified similarity. Finally, the target label is determined according to the calculated similarities.

In the remainder of this study, we do the following: in Section 2, we introduce the extraction of binary target region and ASCs. The main methodology of matching ASCs with the binary target region is presented in Section 3. In Section 4, experiments are conducted on the Moving and Stationary Target Acquisition and Recognition (MSTAR) dataset. Finally, in Section 5, we draw conclusions according to the experimental results, and outline some future work.

## 2. Extraction of Binary Target Region and ASCs

### 2.1. Target Segmentation

We first obtain the binary target region using the target segmentation algorithm. In this study, the detailed target segmentation algorithm consists of the following steps:

(1): Equalize the original image intensities into the range of 0 to 1 by the standard histogram equalization algorithm [32].

(2): Perform mean filtering on the equalized image with a 3 × 3 kernel [32].

(3): Preliminarily segment the “smoothed” image using the normalized threshold of 0.8.

(4): Remove false alarms caused by the noises using the Matlab “bwareaopen” function, which is capable of removing regions with a few pixels.

(5): Perform the binary morphological closing operation [32] to fill the possible holes and connect the target region.

**Figure 1:**
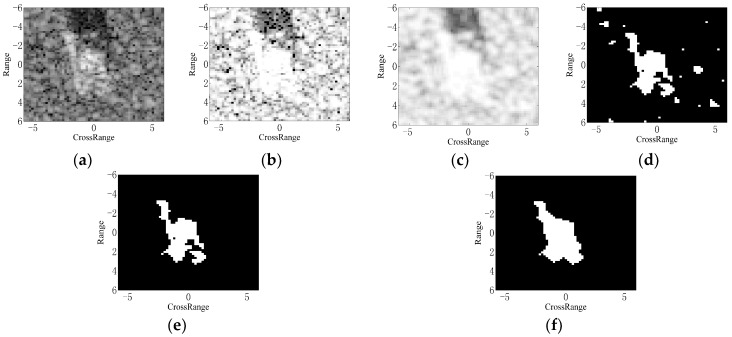
Illustration of the target segmentation algorithm: (**a**) original SAR image of BMP2 tank; (**b**) equalized image; (**c**) smoothed image after mean filtering; (**d**) preliminary segmentation result; (**e**) result after the opening operation; (**f**) result after the closing operation.

**Figure 2:**
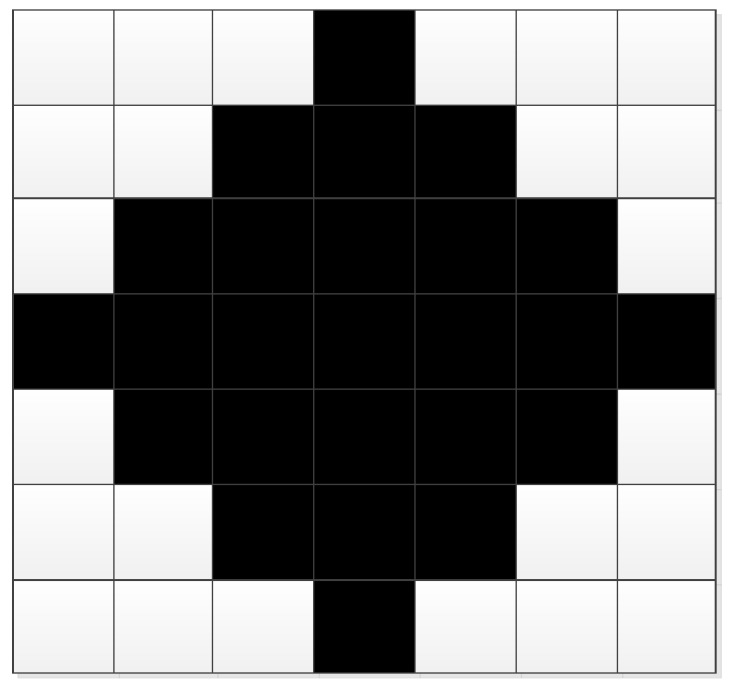
The structuring elements used in the closing operation.

Figure 1 illustrates the implementation of target segmentation with a SAR image of BMP2 tank in the MSTAR dataset shown as Figure 1a. The equalized and smoothed images from Step 2 and Step 3 are displayed in Figure 1b,c, respectively. After the preliminary segmentation, the result is shown in Figure 1d, in which there are some false alarms brought by the noises or clutters. In this step, the threshold is set to be 0.8 mainly according to the repetitive observations at different thresholds, as well as referring to the previous works [22]. The pixel number for the “bwareaopen” function is set to 20; thus, the isolated regions with less than 20 pixels can be eliminated. The result is obtained as Figure 1e. The morphological closing operation is conducted using the 7 × 7 diamond structuring element shown as Figure 2. Finally, the intact binary region is obtained as Figure 1f. The binary target region describes the physical structures and geometrical properties of the target. Actually, it is a continuous region connecting the images of individual scattering centers on the target. From this aspect, the binary target region can be used as the reference for ASC matching.

### 2.2. ASC Extraction

#### 2.2.1. ASC Model

SAR images reflect the target’s electromagnetic characteristics in the high frequency region, which can be quantitively modeled as a summation of local properties, i.e., scattering centers [14]. The target’s backscattering field can be expressed as follows:

(1) $$E(f,\varphi ;\theta )=\sum_{i=1}^{K}E_{i}(f,\varphi ;\theta_{i})$$

In Equation (1), f and φ denotes the frequency and aspect angle, respectively. K is the number of the ASCs in the radar measurement. For a single ASC, its backscattering field can be calculated according to ASC model [14] Equation (2).

(2) $$\begin{array}{ll} E_{i}(f,\varphi ;\theta_{i})= & A_{i}\cdot {\left(j\frac{f}{f_{c}}\right)}^{\alpha_{i}}\cdot \exp(\frac{-j4\pi f}{c}(x_{i}\cos\varphi +y_{i}\sin\varphi ))\\ & \cdot \mathrm{sinc}(\frac{2\pi f}{c}L_{i}\sin(\varphi -\bar{\varphi_{i}}))\cdot \exp(-2\pi f\gamma_{i}\sin\varphi ) \end{array}$$

where c denotes the propagation velocity of electromagnetic wave and $\theta =\{\theta_{i}\}=[A_{i},\alpha_{i},x_{i},y_{i},L_{i},\bar{f_{i}},\gamma_{i}](i=1,\;2,\;\cdots ,\;K)$ represents the attribute set of all the ASCs in a SAR image. In detail, for the ith ASC, $A_{i}$ is the complex amplitude; $\left(x_{i},y_{i}\right)$ denote the spatial positions; $\alpha_{i}$ is the frequency dependence; for a distributed ASC, $L_{i}$ and $\bar{\varphi_{i}}$ represent the length and orientation, respectively; and $\gamma_{i}$ denotes the aspect dependence of a localized ASC.

#### 2.2.2. ASC Extraction Based on Sparse Representation

The characteristics of a single SAR image can be approximated by only a few ASCs. So, the ASCs to be extracted are actually sparse in the model-parameter domain, which discretize the parameter space to form an overcomplete dictionary [33,34]. Therefore, the sparse representation can be employed to estimate the ASC parameters. The ASC model in Equation (1) is first expressed as Equation (3).

(3) s=D(θ)×σ

where s is obtained by reformulating the 2-D measurement E(f,φ;θ) into a vector; D(θ) represents the overcomplete dictionary. In detail, each column of D(θ) stores the vector form of the electromagnetic field of one element in the parameter space θ; σ denotes a sparse vector and each element in it represents the complex amplitude A.

In practical situations, the noises and possible model errors should also be considered. Therefore, Equation (3) is reformulated as follows:

(4) s=D(θ)×σ+n

In Equation (4), n denotes the error term, which is modeled as a zero-mean additive white Gaussian process. Afterwards, the attributes of the ASCs can be estimated as follows:

(5) $$\hat{\sigma }=\underset{\sigma }{\arg\min}{\left\|\sigma \right\|}_{0},\mathrm{s.t.}\;{\left\|s-D(\theta )\times \sigma \right\|}_{2}\leq \varepsilon \;$$

In Equation (5), $\varepsilon ={\left\|n\right\|}_{2}$ represents the noise level; ${\left\|•\right\|}_{0}$ denotes $l_{0}$-norm and $\hat{\sigma }$ is the estimated complex amplitudes with respect to the dictionary D(θ). As a nondeterministic polynomial-time hard (NP-hard) problem, the sparse representation problem in Equation (5) is computationally difficult to solve. As a remedy, some greedy methods, e.g., the orthogonal matching pursuit (OMP), are available [33,34]. Algorithm 1 illustrates the detailed procedure of ASC extraction based on sparse representation.

**Algorithm 1** ASC Extraction based on Sparse Representation**Input:** The vectorized SAR image s, noise level ε, and overcomplete dictionary D(θ). **Initialization:** Initial parameters of the ASCs $\hat{\theta }=\emptyset$, reconstruction error r=s, counter t=1. 1. while ${\left\|r\right\|}_{2}^{2}>\varepsilon$ do 2. Calculate correlation: $C(\theta )=D^{H}(\theta )\times r$, where ${\left(•\right)}^{H}$ represents conjugate transpose. 3. Estimate parameters: ${\hat{\theta }}_{t}=\underset{\theta }{\arg\max}C(\theta )$, $\hat{\theta }=\hat{\theta }\cup {\hat{\theta }}_{t}$. 4. Estimate amplitudes: $\hat{\sigma }=D^{†}(\hat{\theta })\times s$, where ${\left(•\right)}^{†}$ represents the Moore-Penrose pseudo-inverse, $D(\hat{\theta })$ denotes the overcomplete dictionary from the parameter set $\hat{\theta }$. 5. Update residual: $r=s-D(\hat{\theta })\times \hat{\sigma }$. 6. t=t+1 **Output:** The estimated parameters set $\hat{\theta }$.

## 3. Matching ASCs with Binary Target Region

### 3.1. Region Prediction by ASC

**Figure 3:**
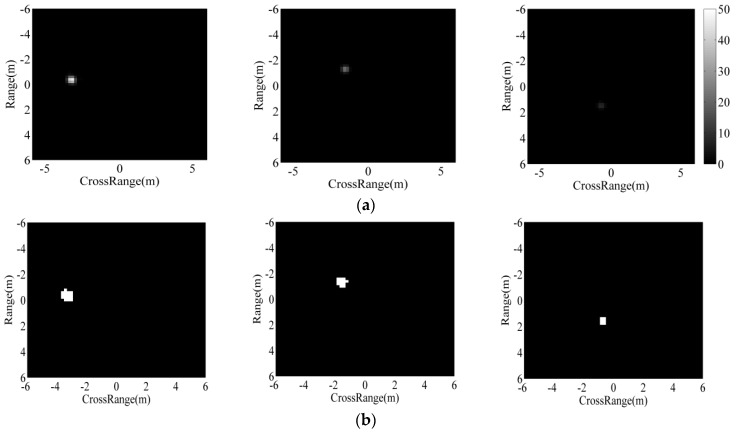
Images and binary regions of ASCs with different amplitudes: (**a**) images; (**b**) binary regions.

**Figure 4:**
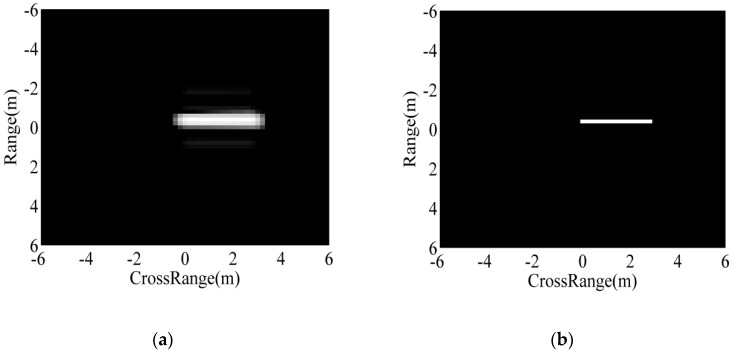
Image and binary region of a distributed ASC: (**a**) image; (**b**) binary region.

**Figure 5:**
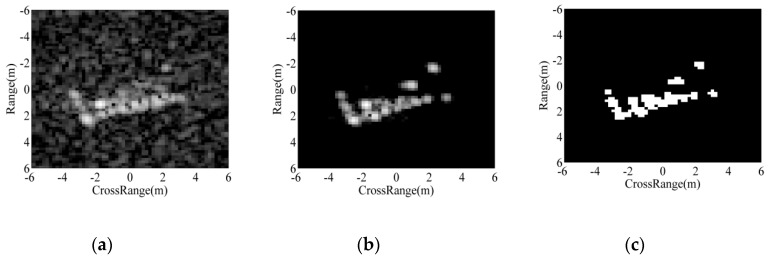
Illustration of ASC extraction and region prediction: (**a**) original image; (**b**) reconstructed image using ASCs; (**c**) overlap of all the predicted regions.

As point features, the matching of two ASC sets is a complex and difficult task, as analyzed in previous research [30]. As a remedy, in this study, the extracted ASCs from the test image are represented as binary regions using a thresholding method. The backscattering field of each ASC is first calculated based on the ASC model in Equation (2). Afterwards, the imaging process is performed to transform the backscattering field to the image domain. In this study, the imaging process is consistent with the MSTAR images including zeropadding, windowing (−35 dB Taylor window), and 2D fast Fourier transform (FFT). The detailed operating parameters of MSTAR SAR images can be referred to [32]. Denoting the maximum intensity of the images from individual ASCs as m, the global threshold for region prediction is set to be m/α, where α is the scale coefficient larger than 1. Figure 3 shows the predicted binary regions of three ASCs with different amplitudes at α=30. The images from ASCs with higher amplitudes tend to have higher pixel intensities, as shown in Figure 3a (from left to right). Their predicted binary regions are shown in Figure 3b, correspondingly. It shows that the stronger ASCs produce binary regions with larger areas. Figure 4 shows the predicted binary region of a distributed ASC. As shown, the length of the distributed ASC can be maintained in the predicted region at the proper threshold. Therefore, the predicted binary region can effectively convey the discriminative attributes of the original ASC, such as spatial positions, relative amplitudes, and lengths. Figure 5 illustrates the target’s image reconstructed by all the extracted ASCs, as well as the predicted regions. Figure 5a shows a SAR image of BMP2 tank. The ASCs of the original image are extracted based on sparse representation and used to reconstruct the target’s image, as shown in Figure 5b. The reconstruction result shows that the extracted ASCs can remove the background interference, while the target’s characteristics can be maintained. Figure 5c shows the overlap of all the predicted regions. Clearly, the predicted regions can convey the geometrical shape and scattering center distribution of the original image.

### 3.2. Region Matching

The predicted regions of individual ASCs are mapped to the target region from the corresponding template samples. It is assumed that the template samples are always obtained in some cooperative conditions. Hence, the template images contain the properties of the intact target at high signal-to-noise ratios (SNR). The detailed steps of the region matching between the test sample and its corresponding template sample can be summarized as follows:

Step 1: The extracted ASCs from the test sample are converted to binary regions according to Section 3.1.

Step 2: Map each of the predicted regions onto the binary target region from the corresponding template sample.

Step 3: The overlapped region between all the predicted regions and the binary target region reflects the correlation between the test and template sample; and the unmatched regions represent their differences. 

**Figure 6:**
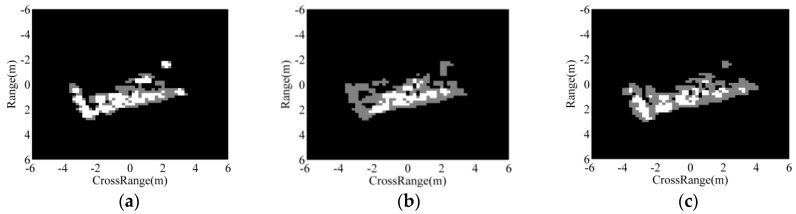
Region matching results between a BMP2 image and three template classes being: (**a**) BMP2; (**b**) T72; (**c**) BTR70.

Figure 6 displays the results of the region matching between the predicted regions of the BMP2 SAR image in Figure 5a and binary target regions from the template samples of BMP2, T72, and BTR70 targets in the MSTAR dataset. The white regions represent the overlap between the predicted regions of the test ASCs and binary target region from the corresponding templates, whereas the grey regions reflect their differences. Clearly, the region overlap with the correct class has a much larger area than those of the incorrect classes. Three scores are defined to evaluate the matching results, as follows.

(6) $$G_{1}=\frac{M}{N},\;G_{2}=\frac{R_{M}}{R_{t}},\;G_{3}=\frac{R_{M}}{R_{N}}\;$$

where N is the number of predicted regions, i.e., the number of all the extracted ASCs. M denotes the number of predicted regions, which are assumed to be matched with the template’s target region. $R_{M}$ denotes the total area of all the matched regions; $R_{N}$ and $R_{t}$ are the areas of all the predicted regions and binary target region, respectively. For a predicted region, it is judged to be matched only if the overlap between itself and the template’s binary region is larger than half of its area.

To combine the advantages of the three scores, a linear fusion algorithm is performed to obtain the overall similarity as Equation (7) [35].

(7) $$S=\omega_{1}G_{1}+\omega_{2}G_{2}+\omega_{3}G_{3}\;$$

where $\omega_{1}$, $\omega_{2}$ and $\omega_{3}$ denote the weights; S represents the fused similarity. With little prior information on which score is more important, equal weights are assigned to the three scores in this study, i.e., $\omega_{1}=\omega_{2}=\omega_{3}=1/3$.

### 3.3. Target Recognition

**Figure 7:**
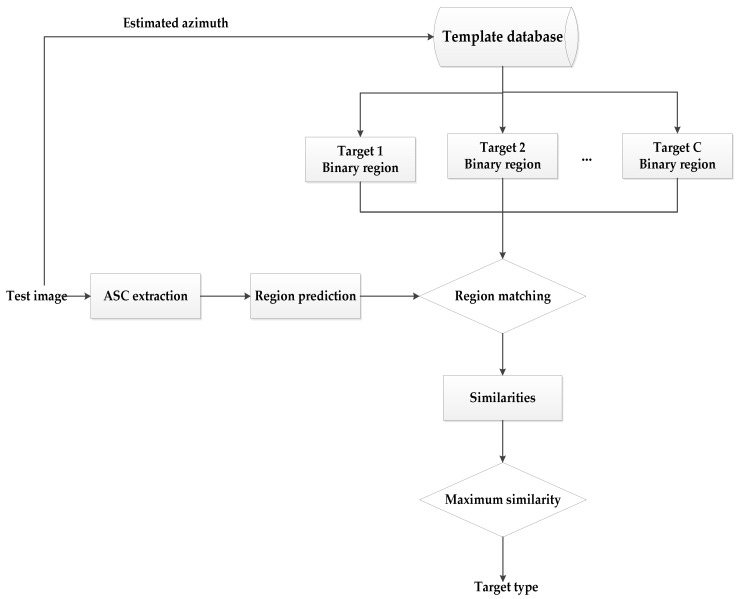
The basic procedure of target recognition.

The proposed matching scheme for the extracted ASCs and binary target region is performed with application to SAR target recognition. The basic procedure of our method is illustrated in Figure 7, which can be summarized as follows.

(1) The ASCs of the test image are estimated and predicted as binary regions.
(2) The azimuth of the test image is estimated to select the corresponding template images.
(3) Extract the binary target regions of all the selected template samples.
(4) Matched the predicted regions to each of the template regions and calculate the similarity.
(5) Decide the target label to be the template class, which achieves the maximum similarity.

Specifically, the azimuth estimation algorithm in [22] is used, which also uses the binary target region. So, it can directly perform on the target region from Section 2 to obtain the estimated azimuth. The estimation precision of the method is about ±5°. Accordingly, in this study, the template samples with azimuths in the interval of [−3°: 1°: 3°] around the estimated one are used as the potential templates. In addition, to overcome the 180° ambiguity, the template selection is performed on the estimated azimuth and its 180° symmetric one, and the average of the similarities from all the candidate template samples is adopted as the final similarity for target recognition. The scale coefficient to determine the global threshold is set as α=30 according to the experimental observations for parameter selection.

## 4. Experiment on MSTAR Dataset

### 4.1. Experimental Setup

#### 4.1.1. MSTAR Dataset

**Figure 8:**
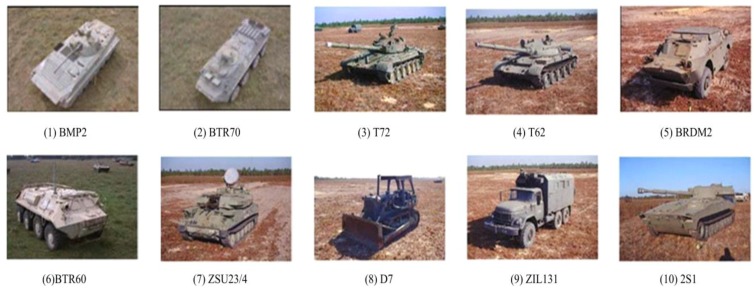
Optic images of the ten targets to be classified.

The widely used benchmark dataset for evaluating SAR ATR methods, i.e., MSTAR dataset, is adopted for experimental evaluation in this paper. The dataset is collected by the Sandia National Laboratory airborne SAR sensor platform, working at X-band with HH polarization. There are ten classes of ground targets with approaching physical sizes, whose names and optic images are presented in Figure 8. The collected SAR images have resolutions of 0.3 m × 0.3 m. The detailed template and test sets are given in Table 1, where samples from 17° depression angle are adopted as the templates, whereas images at 15° are classified.

#### 4.1.2. Reference Methods

In order to reflect the merits of the proposed method, several prevalent SAR target recognition methods are taken as the references, as described in Table 2. For the SVM method, the classifier is performed by the LIBSVM package [36] on the feature vectors extracted by PCA, whose dimensionality is set to be 80 according to previous works [21,24]. In SRC, the OMP algorithm is chosen to resolve the sparse representation tasks of the 80-dimension PCA features. The A-ConvNet is a taken as a representative SAR ATR method using CNN. The designed networks in [25] is used for training and testing based on the original image intensities. The target recognition method based on ASCs in [28] is compared, in which a similarity measure between two ASC sets is formed for target recognition. The region matching method in [3] is also compared. The target region of the test sample is matched with the regions from different classes of templates and the similarities are calculated to determine the target label. All the methods are implemented on a PC with Intel i7 (Intel, Hanoi, Vietnam) 3.4 GHz CPU and 8 GB RAM.

In the following tests, we first perform the experiment to classify the ten targets under SOC. Then, several EOCs including the configuration variants, large depression angle variation, noise contamination, and partial occlusion, are used for further evaluation of the performance of our method. 

### 4.2. Experiment under SOC

At first, the recognition task is conducted under SOC based on the template and test sets in Table 1. Specifically, for BMP2 and T72 with three configurations, only “9563” for BMP2 and “132” for T72 are used in the template samples. Table 3 displays the confusion matrix of our method on the ten targets, in which the percentage of correct classification (PCC) of each class is illustrated. Clearly, the PCCs of these targets are over 96%, and the average PCC is calculated to be 98.34%. Table 4 displays the average PCCs, as well as the time consumption (for classifying one MSTAR image) of all the methods. Our method achieves the highest PCC, indicating its effectiveness under SOC. Although CNN is demonstrated to be effective for SAR ATR, it cannot work well if the training samples are insufficient. In this experimental setup, there are some configuration variants between the template and test sets of BMP2 and T72. As a result, the performance of A-ConvNet cannot rival the proposed method. Compared with the ASC Matching and Region Matching methods, our method performs much better, indicating that the classification scheme in this study can better make use of ASCs and target region to enhance the recognition performance. As for the time consumption, the classifiers like SVM, SRC, and CNN perform more efficiently than the proposed method because of the unified form of the features used in these methods. The ASC matching consumes the most time because it involves complex one-to-one matching between ASC sets. Compared with the region matching method in [3], the proposed method is relatively more efficient. The method in [3] needs to process the region residuals between two binary target regions, which is more time-consuming than the proposed region matching method.

### 4.3. Experiment under EOCs

The template/training samples are usually collected or simulated under some cooperative conditions. EOCs refer to those conditions occurred in the test samples, which are not included in the template/training set, e.g., configuration variants, depression angle variance, noise contamination, etc. To improve the robustness, it is desirable that the ATR methods work robustly under different types of EOCs. In the following paragraphs of this subsection, we evaluate the proposed method under several typical EOCs.

#### 4.3.1. EOC 1-Configuration Variants

**Figure 9:**
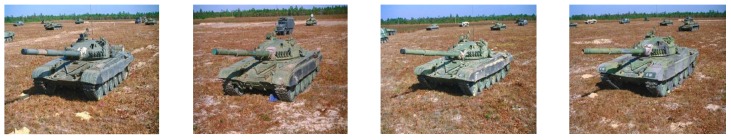
Four different configurations of T72 tank.

The ground military target often has different configurations. Figure 9 shows four different configurations of a T72 tank, which have some locally structurally modifications. In practical applications, the configurations of the test samples may not be included in the template set. Table 5 lists the template and test samples for the experiment under configuration variants. The configurations of BMP2 and T72 to be classified are different to their counterparts in the template sets. Table 6 displays the classification results of different configurations by our method. The test configurations can be recognized with PCCs higher than 96%, and the average PCC is calculated to be 98.64%. Table 7 compares the average PCCs of different methods under configuration variants. The proposed method works most robustly under configuration variants with the highest average PCC. For targets of different configurations, they share similar physical sizes and shape with some local modifications. In this case, the target region and local descriptors can provide more robustness than the global features, like image intensities or PCA features; that’s why the ASC Matching and Region Matching methods outperform the SVM, SRC, and CNN methods in this situation.

#### 4.3.2. EOC 2-Large Depression Angle Variation

**Figure 10:**
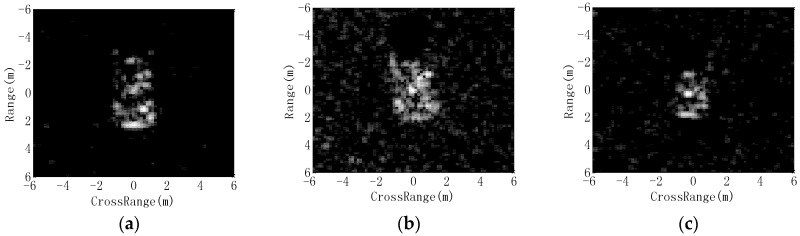
SAR images from depression angles of: (**a**) 17°; (**b**) 30°; (**c**) 45°.

The platform conveying SAR sensors may operate at different heights. Consequently, the depression angle of the measured image is likely to be different with those of the template samples, which are often collected at only one or few depression angles. The template and test sets in the present experiment are showcased in Table 8, where three targets (2S1, BDRM2, and ZSU23/4) are classified. Images at 17° are adopted as the template samples, whereas those at 30° and 45° are classified. SAR images of 2S1 target at 17°, 30° and 45° depression angles are shown in Figure 10, respectively. It shows that the large depression angle variations notably change the appearances and scattering patterns of the target. The results from our method under large depression angle variation are displayed in Table 9. It achieves the average PCCs of 97.80% and 76.16% at 30° and 45° depression angles, respectively. The performances of all the methods under a large depression angle variation are displayed in Table 10. All the PCCs fall sharply at a 45° depression angle, mainly because the test images have significant differences with the training ones, as shown in Figure 10. In the ASC matching method, the similarity evaluation is performed based on the correspondence of two ASC sets. So, some stable ASCs under large depression angle variance still help correct target recognition. Therefore, it achieves a higher average PCC than SVM, SRC, CNN, and region matching methods at a 45° depression angle. In comparison, our method obtains the highest accuracies at both 30° and 45° depression angles, validating its highest robustness in this case. 

#### 4.3.3. EOC 3-Noise Contamination

**Figure 11:**
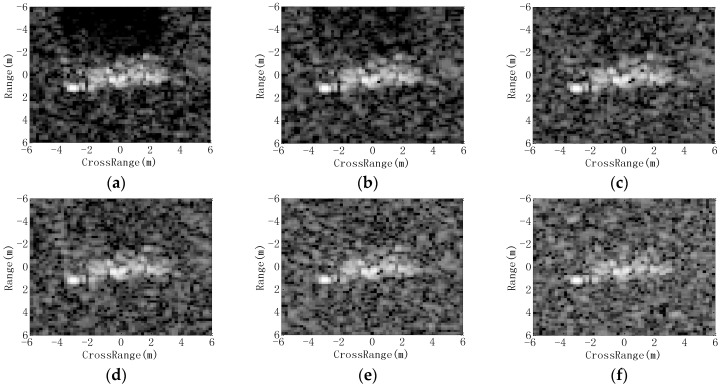
Images with noise addition (SNR): (**a**) original image; (**b**) 10 dB; (**c**) 5 dB; (**d**) 0 dB; (**e**) −5 dB; (**f**) −10 dB.

**Figure 12:**
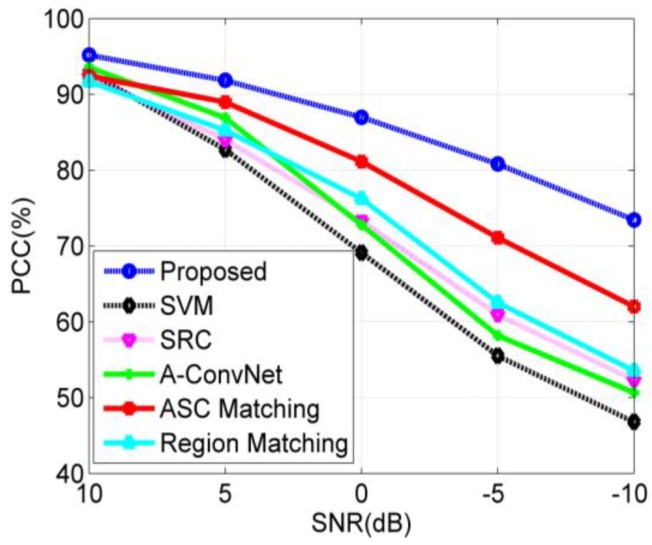
Performance comparison of all the methods under noise contamination.

Noise contamination is a common situation in the practical application of SAR ATR because of the noises from the environment or SAR sensors [37,38,39]. To test the performance of our method under possible noise contamination, we first simulate noisy images by adding Gaussian noises to the test samples in Table 1. In detail, the original SAR image is first transformed into the frequency domain. Afterwards, the complex Gaussian noises are added to the frequency spectrum according to the preset SNR. Finally, the noisy frequency data is transformed back into image domain to obtain the noisy SAR image. Figure 11 shows the noisy SAR images with different levels of noise addition. The average PCCs of all the methods under noise contamination are plotted as Figure 12. As shown, our method achieves the highest PCC at each noise level, indicating the best robustness regarding possible noise contamination. At low SNRs, the intensity distribution changes greatly. However, the ASCs can keep their properties so that they can be precisely extracted by sparse representation. In addition, the target region still contains pixels with higher intensities than the background or shadow pixels. Then, the target region can also be segmented properly. This is also the reason why the ASC Matching method and Region Matching method perform better than SVM, SRC, and CNN.

#### 4.3.4. EOC 4-Partial Occlusion

**Figure 13:**
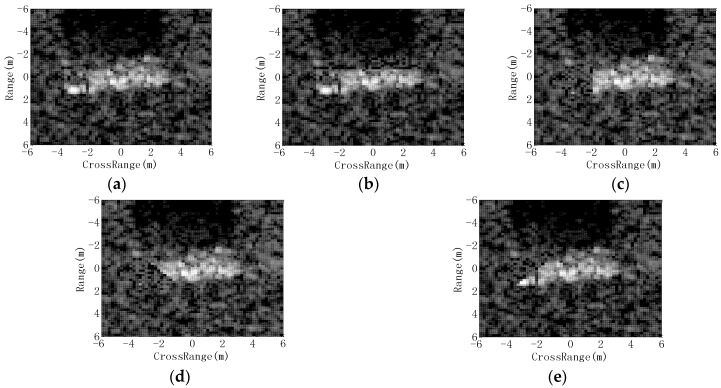
Occluded images at the occlusion level of 20% from different directions being: (**a**) original image; (**b**) direction 1; (**c**) direction 2; (**d**) direction 3; (**e**) direction 4.

**Figure 14:**
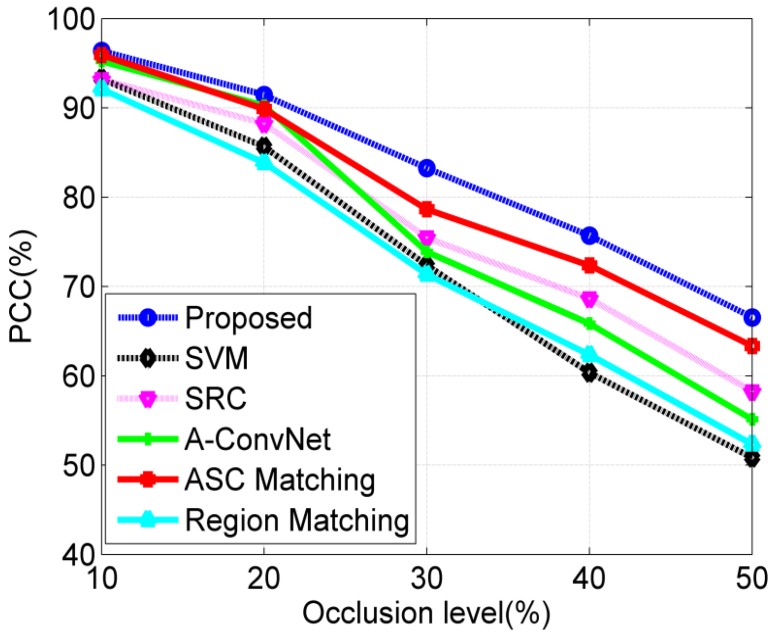
Performance comparison of all the methods under partial occlusion.

In fact, the target may be occluded by the obstacles; thus, a certain proportion of the target may not be captured by SAR sensors. In this experiment, the occluded SAR images are generated as the occlusion model in [40,41]; then, the performance of different methods is evaluated at different occlusion levels. In detail, a certain proportion of the binary target region from the original image is first occluded from different directions. Afterwards, the remaining target region and background are filled with the original pixels, while the occluded region is filled with the randomly picked background pixels. In this way, different levels of partially occluded SAR images from different directions can be generated for target recognition. In Figure 13, some occluded images are shown, in which 20% of the target regions are occluded from different directions. Figure 14 plots the PCCs of all the methods under partial occlusion. Our method obtains the highest PCCs at different occlusion levels, indicating its highest effectiveness under partial occlusion. The predicted regions of ASCs reflect the local features of the target. Although a part of the target is occluded, the remaining parts can still keep stable. In the proposed method, the ASCs are extracted to describe the local characteristics of the original image. The predicted regions can effectively convey the discrimination of the remaining parts, which are not occluded. By matching the predicted regions with the intact target region of the template samples, the proposed method can keep robust under partial occlusion. Similar to the conditions of noise corruption, the ASC Matching, and Region Matching methods perform better than the classifiers performed on the global features, i.e., SVM, SRC and CNN.

## 5. Conclusions

In this study, we propose an effective method for SAR ATR by matching ASCs to binary target region. Instead of directly matching the points features, i.e., ASCs, to the target region, each ASC is predicted as a binary region using a thresholding method. The binary regions of individual ASCs vary in the areas and shapes, which reflect their attributes such as spatial positions, relative amplitudes, and lengths. Afterwards, the predicted regions of the test sample are mapped to the binary target region from the corresponding templates. Finally, a similarity measure is defined according to the region matching results, and the target label is determined according to the highest similarity. The MSTAR dataset is employed for experiments. Based on the experimental results, conclusions are drawn as follows.

(1) The proposed method works effectively for the recognition task of ten targets under SOC with a notably high PCC of 98.34%, which outperforms other state-of-the-art methods.

(2) Under different types of EOCs (including configuration variants, large depression angle variation, noise contamination, and partial occlusion), the proposed performs more robustly than the reference methods owing to the robustness of the region features as well as the designed classification scheme.

(3) Although not superior in efficiency, the higher effectiveness and robustness make the proposed method a potential way to improve the SAR ATR performance in the practical conditions.

Future work is as follows. First, as basic features in the proposed target recognition method, the extraction precision of binary target region and ASCs should be further improved by adopting or developing more robust methods. Some despeckling algorithms [42,43,44] can be first used to improve the quality of the original SAR images before the feature extraction. Second, the similarity measure based on the region matching results should be further improved to enhance the ATR performance, e.g., the adaptive determination of the weights for different scores. Third, the proposed method should be extended to the ensemble SAR ATR system to handle the condition that several targets are contained in a SAR image. Lastly, the proposed method should be tested on other available dataset from the airborne or orbital SAR sensors to further validate its effectiveness and robustness. 

## Figures and Tables

**Table 1:** The template/training and test sets for the experiments.

Target	Serial	Template/Training Set		Test Set	
		Depr.	Number	Depr.	Number
BMP2	9563	17°	233	15°	195
	9566	17°	232	15°	196
	c21	17°	233	15°	196
BTR70	c71	17°	233	15°	196
T72	132	17°	232	15°	196
	812	17°	231	15°	195
	S7	17°	228	15°	191
ZSU23/4	D08	17°	299	15°	274
ZIL131	E12	17°	299	15°	274
T62	A51	17°	299	15°	273
BTR60	k10yt7532	17°	256	15°	195
D7	92v13015	17°	299	15°	274
BDRM2	E71	17°	298	15°	274
2S1	B01	17°	299	15°	274

Depr. is abbreviation of “depression angle”; the picture of each target is given in Figure 8.

**Table 2:** Methods to be compared with the proposed one.

Method	Feature	Classifier	Reference
SVM	Feature vector from PCA	SVM	[20]
SRC	Feature vector from PCA	SRC	[24]
A-ConvNet	Image intensities	CNN	[28]
ASC Matching	ASCs	One-to-one matching	[18]
Region Matching	Binary target region	Region matching	[3]

**Table 3:** Confusion matrix of the proposed method on the ten targets under SOC.

Target	BMP2	BTR70	T72	T62	BDRM2	BTR60	ZSU23/4	D7	ZIL131	2S1	PCC (%)
BMP2	553	6	7	0	0	3	2	0	1	2	96.44
BTR70	0	196	0	0	0	0	0	0	0	0	100
T72	6	4	562	0	0	0	2	5	3	0	96.73
T62	0	0	0	274	0	0	0	0	0	0	100
BDRM2	0	0	0	0	274	0	0	0	0	0	100
BTR60	1	0	0	0	0	193	0	1	0	0	98.94
ZSU23/4	1	0	0	1	1	0	269	0	1	1	98.16
D7	1	0	0	0	0	1	0	271	0	1	98.88
ZIL131	0	2	0	0	1	0	2	0	269	0	98.18
2S1	0	4	0	0	0	1	0	0	0	269	98.18
Average											98.34

PCC: percentage of correction classification.

**Table 4:** Average PCCs of all the methods under the standard operating condition.

Method	Proposed	SVM	SRC	A-ConvNet	ASC Matching	Region Matching
PCC (%)	98.34	95.66	94.68	97.52	95.30	94.68
Time consumption (ms)	75.8	55.3	60.5	63.2	125.3	88.6

**Table 5:** Template and test sets with configuration variants.

	Depr.	BMP2	BDRM2	BTR70	T72
Template set	17°	233 (9563)	298	233	232 (132)
Test set	15°, 17°	428 (9566) 429 (c21)	0	0	426 (812) 573 (A04) 573 (A05) 573 (A07) 567 (A10)

**Table 6:** Classification results of different configurations of BMP2 and T72.

Target	Serial	BMP2	BRDM2	BTR-70	T72	PCC (%)
BMP2	9566	412	11	2	3	96.26
	c21	420	4	2	3	97.90
T72	812	18	1	0	407	95.54
	A04	5	8	0	560	97.73
	A05	1	1	0	571	99.65
	A07	3	2	3	565	98.60
	A10	7	0	2	558	98.41
Average						98.58

**Table 7:** PCCs of all the methods under configuration variants.

Method	Proposed	SVM	SRC	A-ConvNet	ASC Matching	Region Matching
PCC (%)	98.58	95.67	95.44	96.16	97.12	96.55

**Table 8:** Template and test sets with large depression angle variation.

	Depr.	2S1	BDRM2	ZSU23/4
Template set	17°	299	298	299
Test set	30°	288	287	288
	45°	303	303	303

**Table 9:** Classification results of the proposed method at 30° and 45° depression angles.

Depr.	Target	Classification Results			PCC (%)	Average (%)
		2S1	BDRM2	ZSU23/4		
30°	2S1	278	6	4	96.53	97.68
	BDRM2	1	285	1	99.30	
	ZSU23/4	4	4	280	97.22	
45°	2S1	229	48	26	75.58	75.82
	BDRM2	10	242	51	79.87	
	ZSU23/4	54	31	218	71.95	

**Table 10:** PCCs of all the methods at 30° and 45° depression angles.

Method	PCC (%)	
	30°	45°
Proposed	97.68	75.82
SVM	96.87	65.05
SRC	96.24	64.32
A-ConvNet	97.16	66.27
ASC Matching	96.56	71.35
Region Matching	95.82	64.72
